# Balanced Polymorphism at the *Pgm-1* Locus of the Pompeii Worm *Alvinella pompejana* and Its Variant Adaptability Is Only Governed by Two QE Mutations at Linked Sites

**DOI:** 10.3390/genes13020206

**Published:** 2022-01-24

**Authors:** Alexis Bioy, Anne-Sophie Le Port, Emeline Sabourin, Marie Verheye, Patrice Piccino, Baptiste Faure, Stéphane Hourdez, Jean Mary, Didier Jollivet

**Affiliations:** 1 Adaptation et Diversité en Milieu Marin, Equipe ABICE, Station Biologique de Roscoff, UMR 7144 Sorbonne Université-CNRS, 29688 Roscoff, France; alexis.bioy@hotmail.fr (A.B.); asleport@sb-roscoff.fr (A.-S.L.P.); jmary@sb-roscoff.fr (J.M.); 2 Institut de Recherche Pour la Conservation des Zones Humides Méditerranéennes, Tour du Varlat, Le Sambuc, 13200 Arles, France; emeline.sabourin@gmail.com; 3 Royal Belgian Institute of Natural Sciences, Rue Vautier 29, 1000 Brussels, Belgium; mverheye@naturalsciences.be; 4 Lycée Lakanal, 3 Avenue du Président Franklin Roosevelt, 92330 Sceaux, France; patricepiccino@yahoo.fr; 5 Biotope—Agence Nord-Littoral, ZA de la Maie, Avenue de l’Europe, 62720 Rinxent, France; bfaure@biotope.fr; 6 Observatoire Oceanologique de Banyuls-sur-Mer, UMR 8222 CNRS-SU, 1 Avenue Pierre Fabre, 66650 Banyuls-sur-Mer, France; hourdez@obs-banyuls.fr

**Keywords:** phosphoglucomutase, balancing selection, thermal stability, gene, adaptive mutations, Alvinellidae

## Abstract

The polychaete *Alvinella pompejana* lives exclusively on the walls of deep-sea hydrothermal chimneys along the East Pacific Rise (EPR), and displays specific adaptations to withstand the high temperatures and hypoxia associated with this highly variable habitat. Previous studies have revealed the existence of a balanced polymorphism on the enzyme phosphoglucomutase associated with thermal variations, where allozymes 90 and 100 exhibit different optimal activities and thermostabilities. Exploration of the mutational landscape of phosphoglucomutase 1 revealed the maintenance of four highly divergent allelic lineages encoding the three most frequent electromorphs over the geographic range of *A. pompejana*. This polymorphism is only governed by two linked amino acid replacements, located in exon 3 (E155Q and E190Q). A two-niche model of selection, including ‘cold’ and ‘hot’ conditions, represents the most likely scenario for the long-term persistence of these isoforms. Using directed mutagenesis and the expression of the three recombinant variants allowed us to test the additive effect of these two mutations on the biochemical properties of this enzyme. Our results are coherent with those previously obtained from native proteins, and reveal a thermodynamic trade-off between protein thermostability and catalysis, which is likely to have maintained these functional phenotypes prior to the geographic separation of populations across the Equator about 1.2 million years ago.

## 1. Introduction

A central goal in evolutionary biology is to understand the origin and maintenance of polymorphisms sculpted by natural selection and, more specifically, how the mean phenotype of a population evolves under heterogeneous and/or changing conditions [1]. As a consequence, many studies have investigated the maintenance of enzyme polymorphisms by selective processes for species exposed to environmental gradients such as temperature, salinity, or desiccation [2]. A few decades ago, a series of enzymes interacting in the glycolytic cycle, mostly associated with isomerase and mutase functions, such as phosphoglucose isomerase (PGI), mannose phosphate isomerase (MPI), and phosphoglucomutase (PGM), were shown to display isoforms that may be the subject of natural selection, leading to habitat-driven differentiation in populations according to temperature, wave action, or metallic pollution [2,3,4,5,6,7,8,9,10]. According to Eanes [11], such branch-point enzymes—which are positioned at the crossroad of metabolic pathways—are likely to be the target of natural selection, as they can orient pathway fluxes according to their protein variation. Among these, alleles encoding the enzyme phosphoglucomutase have been widely studied, with the aim of testing the hypothesis of differential and/or balancing selection. This was mainly achieved by looking at allele [3,4,12] and heterozygote [13] frequencies in populations, as well as assessing either the fitness of individuals carrying alleles suspected to be locally advantageous along latitudinal clines [14] or the kinetic properties of the enzyme isoforms themselves [6,12].

Due to their tremendous thermal variability, caused by the chaotic mixing of cold sea water and hot fluids, hydrothermal vents represent an ideal model for testing the effect of frequent, and unpredictable, spatial and temporal changes of habitats on adaptive enzyme polymorphisms. First, both the fragmentation and instability of the vent discharge likely promote highly dynamic meta-populations with recurrent local extinctions and associated bottlenecks [15,16,17]. Long-term oscillations of heat convection beneath the ridge lead to the displacement of the hydrothermal activity along the rift, generating the emergence of new vent sites more or less close to older ones that became extinct, allowing for their rapid recolonization [16,18]. Such dynamics should have severe implications in reducing the genetic diversity of vent species. Second, variations in temperature, sulphide concentrations, and oxygen concentrations over short periods of time (often ranging from minutes to hours) [19,20] are likely to affect the respiratory, nutritional, and reproductive physiologies of animals living in such places [21]. Animals must be able to feed under high temperatures to fuel their symbionts and respire under cold conditions to obtain oxygen. These temporal fluctuations of the vent conditions at the individual scale represents a selective constraint that should promote the maintenance of both thermostable and cold-functioning enzyme alleles through mean overdominance until the exploration of the mutational landscape of a given enzyme leads to the emergence of a highly plastic allelic isoform, and thus enzyme monomorphism. However, the hydrothermal environment is also highly fragmented and heterogeneous, according to the mineral composition of the oceanic crust through which the super-heated fluid moves prior to be expulsed above the seafloor [22]. As a consequence, vent fields often display a mosaic of edifices of different ages [23,24], whose mean age and size distribution is dictated by the frequency of tectonic and volcanic events, as well as the dynamics of the heat convection beneath oceanic ridges [25,26,27]. Vent edifices usually cool down with age until they turn off. Depending on the age of the edifice, populations of vent species are thus spatially subjected to a wide variety of vent conditions, which represents an ecological basis for diversifying selection. As most vent species are quite thermotolerant, such a mosaic of habitats is likely to favour thermostable alleles in newly formed edifices and cold functioning ones in older habitats, the fate of a given allele depending on the relative proportions of these habitats.

The polychaete *Alvinella pompejana*, which lives on the hottest part of the hydrothermal-vent environment [28,29] can withstand temperatures up to 50 °C [30]. This tube-dwelling worm lives on the walls of hydrothermal-vent chimneys from a latitude of 23° N on the East Pacific Rise (EPR) to 38° S on the Pacific Antarctic Ridge (PAR) [31]. It has developed peculiar physiological adaptations in order to colonize this hostile habitat [32,33]. Earlier genetic studies have shown that *A. pompejana* exhibits quite an unusually high level of genetic diversity [31,34,35] with a non-negligible number of bi-allelic enzyme loci with equally frequent alleles, some of which display different thermal stabilities [36]. Among them, the enzyme phosphoglucomutase (PGM-1) possesses four distinct isoforms. Allozymes 90 and 100 have frequencies of approximately 35% and 60%, respectively, in populations of the northern EPR, while the two other isoforms (112 and 78) are rather rare, accounting for the remaining 5%. Although the frequency of allozyme 90 remains constant over the species range, Plouviez et al. [35] have shown that allozymes 78 and 100 display an abrupt clinal distribution across the Equator, with allozyme 78 becoming the most frequent allele in the southern EPR. Bi-allelism was, thus, preserved all along the EPR despite population isolation, recurrent extinction/recolonizations, and a long history of divergence across the Equatorial barrier.

In addition, significant genetic differentiation has been observed between *A. pompejana* populations living in contrasting microhabitats, especially when comparing newly formed ‘still hot’ chimneys (i.e., ‘hot’ niche) to older and colder edifices (i.e., ‘cold’ niche). The frequency of allele 90 is, indeed, positively correlated with mean temperature at the opening of *Alvinella* tubes and increases in the ‘hot’ compared to the ‘cold’ habitat, suggesting that this locus is under diversifying selection [12]. In vitro experiments on enzyme stability and optima have strengthened this view. They have shown that allele 90 is more thermostable and more active at higher temperatures than allele 100 and, thus, is probably favoured in the ‘hot’ habitat.

Although whole-length *Pgm-1* sequences have now been obtained for a large panel of metazoan species, very few studies have been conducted at the population level, most of which involved bacterial strains. While this enzyme has been extensively studied from the 1970s to 1990s for adaptive purposes, only few studies have examined the relationship between nonsynonymous changes at the gene level and the subsequent enzymatic performance of alternate isoforms (see, e.g., [14,37] for the correspondence between allelism, enzyme thermal resistance, and glycogen storage in *Drosophila*). In this paper, we report a possible case of long-term balancing selection at an enzyme locus where alleles can be maintained by varying selection between two niches, possibly helped by fluctuations in the relative proportions of the two niches over space and time. Most of the documented cases for the long-term persistence of alleles by balancing selection and trans-species polymorphism come from studies dealing with negative frequency-dependent selection at immune and sex-determination genes [38,39]. This raises questions about how a chaotic and highly fluctuating two-niche system can promote balancing selection at key branch-point enzymes. The aim of this work was, therefore, to identify the mutations at the basis of the enzyme polymorphism of PGM-1 in the Pompeii worm and to evaluate the effects of these mutations on the thermostability and catalytic efficiency of the enzyme to test whether a trade-off between these two processes is likely to explain the maintenance of the different isoforms. In parallel, we explored the mutational landscape of the gene to search for traces of balancing selection in the vicinity of the amino acid substitutions that led to these isoforms and assess their long-term duration. Finally, we also examined the evolutionary mechanisms: (1) fitness cost to the colonization of newly ‘hot’ habitats; (2) overdominance associated with the fluctuations of the vent discharge, and (3) the dynamics of hot vs cold habitats (i.e., the two-niches model hypothesis), by which these isoforms could have been maintained in natural populations by analyzing the fecundity of females carrying the most thermostable isoform, and by using MSMS simulations implementing selection across a barrier to gene flow.

## 2. Materials and Methods

### 2.1. Animal Sampling

**Figure 1:**
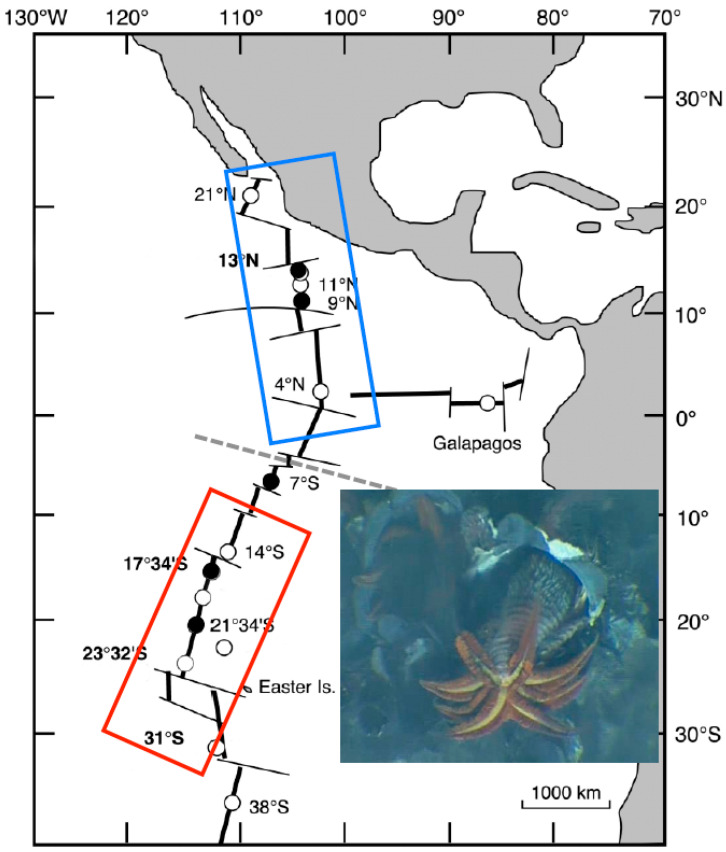
Species range of the Pompeii worm *Alvinella pompejana* along the East Pacific Rise (EPR). Dashed line indicates the presence of the Equatorial barrier to gene flow depicted by Plouviez et al. (2009, 2010) [35,40]. Blue and red boxes correspond to the northern and southern metapopulations of the worm. Dots represent localities where the worms are present and black ones, the localities from which the worms have been sampled to perform the present study. Inset includes a close 10 cm^2^ view of *A. pompejana* moving outside its tube on the wall of a hydrothermal vent chimney (IFREMER copyright). The photograph shows the anterior part of the worm with its branchial crown and its epibiotic filamentous bacteria located dorsally.

Specimens of *A. pompejana* were collected with the remotely operated vehicle (ROV) Victor 6000 and the deep-sea manned submersible Nautile during the cruises Phare 2002, Biospeedo 2004, and Mescal 2010 on board the research vessel L’Atalante. Animals were sampled from targeted sites located on the northern EPR (NEPR) and southern EPR (SEPR; see Figure 1) over chimneys of different ages ranging from newly formed ‘hot’ diffusors to large black ‘smokers’, at which the thermal and chemical conditions were highly contrasting [24].

In order to test an earlier hypothesis [12], postulating that individuals carrying genotypes favoured during the colonization of newly formed still ‘hot’ chimneys may be counter-selected by a lower reproductive fitness under cooler conditions (i.e., trade-off between colonization ability and reproduction), we examined the relationship between *Pgm-1* genotypes and the fecundity of females. To perform this test, the size of animals was estimated from the width at the S4 setigerous segment, and sexes were determined based on the presence of either a genital pore in females or a pair of sexual tentacles in males [41]. Mature females collected from both sides of the Equator were dissected to estimate their fecundity per size unit and genotyped at the *Pgm-1* locus. For each female, the coelomic fluid containing oocytes was carefully removed and re-suspended in 50 mL of a solution of borate-buffered 3% formalin in seawater. Oocytes were counted following the method previously described by Faure et al. [42], and one-way ANOVA was performed on size-corrected female fecundities, according to the genotype, using the Jamovi software (https://www.jamovi.org (12 March 2020)).

### 2.2. Identification and Characterization of the AP-Pgm-1 Gene

#### 2.2.1. Sequencing of *AP-Pgm-1* cDNA Using Homozygous Individuals

Based on allozyme genotypes, eight homozygous individuals carrying alleles 78, 90, and 100 were selected for RNA extractions. Total RNAs were extracted with Tri-Reagent (Sigma-Aldrich, St Quentin-Fallavier, France), following the manufacturer’s instructions, and a classical chloroform extraction protocol. Both the quantity and quality of RNA were assessed using a Nanodrop ND-1000 spectrophotometer (Nanodrop Technologies, Villebon-sur-Yvette, France). Five μg of total RNAs were reverse transcribed with M-MLV reverse transcriptase (Promega, Charbonnières-les-bains, France), anchor-oligo(dT) primer (Table S1), and random hexamers (Promega). Reverse anchor and forward-nested degenerate *Pgm* primers derived from the oyster *Crassostrea gigas* and human *Pgm-1* sequences were then used to perform a preliminary amplification of cDNA fragments (see Table S1). PCR-products containing *Pgm-1* cDNA candidates were then cloned using a TOPO TA Cloning kit (Invitrogen, Villebon-sur-Yvette, France) and sequenced on an ABI 3130 sequencer using the BigDye v.3.1 (PerkinElmer, Villebon-sur-Yvette, France) terminator chemistry, following the manufacturer’s protocol. Sequences of clones containing appropriate *Pgm-1* cDNA fragments were then aligned to reconstruct a series of nearly complete *AP-Pgm-1* cDNA (i.e., only lacking a small part of the 5′-end of the coding sequence).

#### 2.2.2. Sequencing the *AP-Pgm-1* Gene with a Series of Specific Exonic Primers

**Figure 2:**
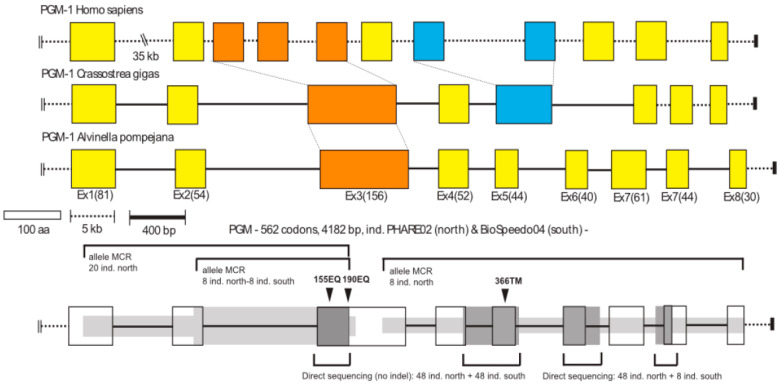
Gene organization of the *Pgm-1* gene for human (*Homo sapiens*), the oyster (*Crassostrea gigas*) and the worm *A. pompejana*. Scale bars indicate the size of exons (white box in terms of amino-acid residues) and introns (dashed and black bars: dashed scaled bar was more appropriate for the human gene which has introns of greater size). Values in parentheses correspond to the exact number of amino acid residues per exon in the worm *A. pompejana*. The dark and light grey zones associated with the *AP-Pgm1* gene (bottom of the figure) highlight the different regions of the gene which has been sequenced for the study with the Mark, Cloning, Recapture (MCR) method (light grey) or direct sequencing (dark grey) together with the number of individuals used. Black arrows indicate the position of the main amino-acid substitutions onto the gene.

Using gDNA, specific reverse primers (Table S1) were also used to amplify the 5′ portion of the gene by directional genome walking using PCR [43]. A series of specific primers were designed based on previously obtained cDNA sequences (see Table S1) in order to amplify both exon- and intron-containing portions of the gene using the same gDNA from the eight homozygous individuals. Fragments of the gene were obtained using pairs of the least distant forward and reverse primers containing a 6-bp individual identifier (barcode). PCR amplifications were performed in a 25 μL PCR reaction volume comprising 1× buffer (supplied by manufacturer), 2 mM MgCl_2_, 0.25 mM of each dNTP, 0.4 μM of each primer, and 0.5 U of Taq polymerase (Thermoprime plus). The PCR profile included a first denaturation step at 94 °C for 4 min, followed by 30 cycles at 94 °C for 30 s, 60 °C for 30 s, and 72 °C for 2 min, then a final extension at 72 °C for 10 min. All barcoded PCR products were cloned following the Molecular Cloning Recapture (MCR) method developed by Bierne et al. [44] and sequenced on an ABI 3130 sequencer with the protocol used previously. Alignments of the sequenced fragments allowed us to reconstruct a complete sequence of the *AP-Pgm-1* gene (Accession Number MN218831), its associated cDNA sequence, and three native consensus cDNA for the three isoforms (Accession Numbers MN218832–MN218839). The analysis of this initial cDNA alignment provided initial information on polymorphic sites between the three distinct alleles along the gene (see Figure 2).

#### 2.2.3. Correspondence between Allozymes and Non-Synonymous Mutations of *AP-Pgm-1*

To examine the correspondence between the only two diagnostic polymorphic nonsynonymous EQ mutations found at exon 3 and allozymes 78, 90, and 100, a total of 220 individuals were genotyped on the 350 bp fragment of the *Pgm-1* exon 3 containing these sites. PGM-1 allozymes were first screened for each individual by electrophoresis on 12% starch-gel at 4 °C (100 V, 80 mA, 4 h) with the Tris-citrate pH 8.0 buffer system, following the procedure described by Piccino [12]. The 350 bp exon 3 fragment was then amplified by PCR for the same individuals, following gDNA extraction using the CTAB/PVP procedure described by Plouviez [40]. PCR amplifications were conducted using a specific primer pair (see Table S1) with a first denaturation step at 94 °C for 4 min, followed by 40 cycles at 94 °C for 30 s, 60 °C for 30 s, and 72 °C for 20 s, then a final extension at 72 °C for 2 min. PCR products were first double-digested on 33 individuals using the enzymes *Fai I* (targeting the first substitution site) and *Bsg I* (targeting the second site) as an initial test, and then sequenced without cloning using an ABI 3130 automatic sequencer with the BigDye v.3.1 (PerkinElmer) terminator chemistry after an ExoSAP-IT purification.

Forward and reverse sequences were proof-read in CodonCode Aligner to check for the occurrence of single (homozygotes) or double (heterozygotes) peaks at the two polymorphic sites. The allele alignment was deposited in Genbank (accession numbers MN218918–MN219291). Linkage disequilibrium between genotypes EE, EQ, and QE and allozymes 78, 90, and 100 was tested using Linkdis function [45] of the Genetix v.4.05 software [46]. The double mutation scoring among individuals allowed us to then estimate heterozygote excesses or deficiencies within populations. Departures to HWE were tested with 1000 permutations of alleles between genotypes using the same software. The exon 3 allele alignment was also used to reconstruct an allelic network, using Network 4.5.1.0 [47], in order to examine gene flow across the equatorial barrier between populations at this locus.

#### 2.2.4. Examining the Synonymous and Non-Synonymous Changes along *AP-Pgm-1*

Nucleotidic diversities were punctually assessed along the gene by combining direct sequencing and the MCR method, considering individuals from each side of the EPR (see Figure 2). These regions included exon 1, exons 4 to 5, the end of exon 7, and the beginning of exon 9 (Accession Numbers MN218840–MN218917 for exon1, Accession Numbers MN219292–MN219356 for exons 4 and 5, see Figure 2). In addition, a fragment containing the whole intron 2 and the beginning of exon 3, where the two diagnostic EQ mutations are located (1110 bp), was also sequenced using the MCR method [44]. This allowed us to test whether mutation hotspots occur around these two EQ sites and to estimate allele divergence (Accession Numbers MN219357–MN219404). In the MCR sequence sets, the number of retrieved alleles greatly varied between the different parts of the gene according to the sequencing efficiency and/or cloning success. Artefactual singletons due to the MCR method were removed by comparing singleton rates between the MCR and direct sequencing data sets on the same fragments.

Haplotype diversity (Hd), nucleotide diversity (π) and its synonymous and non-synonymous components (π_S_ and π_N_), and Watterson’s theta (θ_W_) were then examined together with deviations to neutral evolution (Tajima’s D and Fu & Li’s F statistics) for both the northern and southern EPR individuals along the gene, using the DNAsp 4.10.3 software [48] with a sliding window (size = 100 and step = 25). These basic genetic parameters were then compared with the critical values associated with sample size and neutral coalescent simulations, as implemented in the same software. Linkage disequilibrium between segregating sites and recombination among alleles were estimated by calculating the *ZnS* statistics [49] together with the minimum number of recombination events (*Rm* [50]). The number of significant associations between linked sites was evaluated following a Fisher’s exact test and a Bonferroni correction, implemented in DNAsp 4.10.3. The occurrence of recombinants was also checked using automated RDP and bootscan packages of RDP v.3.44 [51], and recombination hotspots were searched by examining the population recombination rate parameter (4Ne.r) along the gene (-recomb and -hotspot outputs) for both the northern and southern populations using Phase 2.1.1 software [52]. Genetic differentiation and allele divergence between the southern and northern parts of the EPR were estimated by calculating Fst and Dxy in DNAsp 4.10.3. Genetic differentiation was tested using 1000 permutations of the sequence data sets using the randomization test developed by Hudson [53]. Finally, the intron 2-exon 3 alignment (1110 bp) was used to reconstruct a coalescent tree of *AP-Pgm-1* alleles to more specifically evaluate both the intra-locus recombination and allele divergence near the two EQ non-synonymous polymorphic sites. The evolutionary history of alleles was inferred using the Minimum Evolution method implemented in MEGA7 [54] using the NJ algorithm for the initial tree, pairwise deletion of ambiguous sites, and the close-neighbour-interchange (CNI) algorithm. Evolutionary distances were computed using the Maximum Composite Likelihood method.

### 2.3. Coalescent Simulations Using Models of Selection

Coalescent simulations under a divergence model with asymmetrical migration rates between two populations (pop_1 -> 2_: 2N.m = 1 and pop_2 -> 1_: 2N.m = 0.1, N = 1000 simulations with Ne = 50,000) were performed using MSMS v3.2 software [55]. Two different hypotheses of balancing selection were evaluated: (1) over-dominance, with genotype selection coefficients S_aA_ = 500 and S_AA_ = 1 where S represents *N_e_.s*; and (2) habitat-dependent selection, with four populations and two habitats, where S_AA_ = 1000, S_aA_ = 500, and S_aa_ = 0 in the first habitat and S_AA_ = 0, S_aA_ = 500, and S_aa_ = 1000 in the second habitat. Each set of simulations, including the null hypothesis of asymmetrical migration without selection, were run with two recombination rates (*N_e_.r* = 1 or 100). Population parameters, including gene diversities (θ_W_, π), and Tajima’s D within each deme and Fst between demes were estimated using the pylibseq 0.2.3 libraries [56] and a home-made python script.

### 2.4. Functional and Structural Analysis of PGM-1 Recombinant Isoforms

#### 2.4.1. Plasmid Construction for Enzyme Expression

Full-length *AP-Pgm* cDNA was obtained from two homozygous individuals: 100/100 (EE) and 90/90 (EQ). RT-PCR was conducted using a ClonTech SMARTer Race cDNA amplification kit, following the manufacturer’s instructions and using AP-PGMex11 reverse primer (see Table S1). These cDNAs were then used as a target to specifically amplify the complete coding sequence, with primers containing cutting sites to be inserted in either Pet20b or PetDuet expression vectors (Table S1). Amplified coding sequences were double digested with either the enzymes *BamHI*/*NotI* or *AseI*/*XhoI*, in a 25 μL volume containing a restriction buffer, the enzymes, and 1% BSA. The restriction products were then ligated in the appropriate expression vector after purification with a Nucleospin Gel extraction Clean up column (Macherey Nagel, Hoerdt, France) and cloned into BL21DE3 *Escherichia coli* cells amenable for IPTG induction and protein expression.

#### 2.4.2. Directed Mutagenesis

Using full-length cDNA with the double mutation EE as a template, mutants _155_EQ and _190_EQ were produced by directed mutagenesis following the PCR protocol of Reikofski and Tao [57]. First, amplifications were conducted in a 50 µL reaction volume containing 1× Pfu buffer containing MgCl_2_, 0.25 mM of each dNTP, 0.5 μM of each primer (petDuet and mutated primer), and 0.5 U of the proof-reading *Pfu* polymerase (Promega) with 30 cycles of 94 °C for 30 s, 60 °C for 30 s, and 72 °C for 3 min. Then, the two regions of the mutated cDNA were joined following PCR amplification without primers, mixing the two previous PCR-products (1:1) under the same conditions and with a final elongation step of 10 min. cDNAs containing the mutated sites _155_E -> Q and _190_E -> Q and the native _155_E_190_E cDNA were then sequenced using an ABI 3130 sequencer with the BigDye v.3.1 (Perkin Elmer) terminator chemistry to verify the sequences before protein expression.

#### 2.4.3. Protein Expression and Purification

*E. coli* (BL21DE3) with the recombinant pETduet plasmid containing either native or mutated *Pgm-1* cDNA sequences were grown in LB medium supplied with 100 µg/mL ampicillin at 37 °C until they reached an absorbance of 0.6 at 600 nm. Protein expression was induced by adding 1 mM IPTG to the medium and kept at 37 °C under shaking for 4 h. Cells were then harvested by centrifugation (4 °C/15,000× *g*/5 min), and the pellets were resuspended in a binding buffer (20 mM Tris-HCl, pH 6.5; 500 mM NaCl; 5 mM imidazole), disrupted by French Press at 1.6 kbar. After removing cell debris by centrifugation (15,000× *g*/4 °C/60 min), the supernatants (1 μg/mL of lysate) were treated with DNAse I (Eurogentec, Angers, France) for 1 h on ice. A first purification step was performed using immobilized metal affinity chromatography with a His-bind resin column (His-Bond kit, Novagen^®^, Molsheim, France) to recover PGM-1 variants. Protein binding with 5 mM and 60 mM imidazole and final elution of allozymes with 1 M imidazole were performed, following a classical chromatography protocol (pH 6.5). The eluted fractions were concentrated using 30 kDa molecular cut-off Amicon-Ultra (Millipore^TM^). A second purification step was performed by size-exclusion chromatography (SEC) with a Superdex 75 column (1 × 30; GE Healthcare, Velizy, France) at a flow rate of 0.5 mL/min, monitored at 280 nm using 25 mM Na_2_HPO_4_/NaH_2_PO_4_, pH 6.5. The purity of proteins was checked by SDS-PAGE stained with Coomassie brilliant blue, before being kept at 4 °C in elution buffer supplemented with dithiothreitol (DTT, 10 mM) until use for enzyme assays. The protein concentrations were measured by absorption at 280 nm, with the theoretical coefficient of 48,820 M^−1^.cm^−1^, as calculated using the ExPASy-ProtParam tool (http://web.expasy.org/protparam/ (20 June 2019)).

#### 2.4.4. Enzyme Activity Assay

PGM activities were assayed by coupling the formation of α-D-glucose 6-phosphate (G6P) from α-D-glucose 1-phosphate (G1P) to NADPH formation using glucose 6-phosphate dehydrogenase (G6PD) as a relay enzyme. The reaction mixture contained 50 mM Tris-HCl, pH 7.4; 0.5 M MgCl_2_; 1.2 mM NADP; and 0.1 µM G6PD. The recombinant PGMs were used at the following concentrations: [PGM_78_] = 0.9 µM, [PGM_90_] = 4 µM, and [PGM_100_] = 0.6 µM. The concentration of the substrate (G1P) was varied from 0.2 to 60 mM, to determine the kinetic constants *K_m_* and *V_max_* using a Lineweaver–Burk plot.

#### 2.4.5. Thermal Inactivation

The purified PGM activities were measured at 37 °C and at 340 nm using an UVmc^2^ spectrophotometer (Safas, Monaco) after a 30-min incubation at challenge temperatures ranging from 5 to 60 °C. Activities were then normalized as the percentage of residual activity when compared to the same sample kept on ice. A theoretical curve with the following equation was fitted to each experimental data set using a nonlinear curve fit algorithm (Kaleidagraph 4.5.0, Synergy Software, Reading, PA, USA) [58]:

(1) $$y=\frac{\left(y_{N}+m_{N}\cdot \;T\right)+\left(y_{D}+m_{D}\cdot T\right)\cdot \exp\left(\frac{m\left(T-T_{m}\right)}{RT}\right)}{1+\exp\left(\frac{m\left(T-T_{m}\right)}{RT}\right)},$$

where *y* is the residual activity; *y_N_*, *m_N_*, *y_D_*, and *m_D_* are parameters characterizing the activity of the native enzyme (*N*) and its denatured form (*D*), respectively; *m* characterizes the transition between the native and denatured forms; *R* is the universal gas constant; *T* is the absolute temperature, and *T_m_* the absolute temperature of half-denaturation (i.e., the temperature at which the activity of the enzyme is reduced by half).

### 2.5. Guanidinium Chloride-Induced Unfolding of PGM Isoforms

Unfolding of PGM isoforms was induced by guanidinium chloride (*GdmCl*) in a 25 mM sodium phosphate (pH 6.5) and 200 mM NaCl buffer. Proteins (12 μM) were incubated with increasing concentrations of *GdmCl* from 0 to 5 M, for 30 min at 20 °C, and their intrinsic fluorescence emission was determined at 324 nm under excitation at 290 nm using a Safas Xenius spectrofluorimeter (Safas, Monaco). The *GdmCl* concentration was determined by refractive index measurements [59]. Biphasic states of protein denaturation, with an intermediate state (I) between native (N) and unfolded (U) states according to the equilibrium N↔I↔U, were treated as follows. We assumed that each transition (N↔I and I↔U) followed a two-state model of denaturation. The denatured protein fraction for each transition—*f*(I) for transition (N↔I) and *f*(II) for transition (I↔U)—was determined by resolving the two following equations:

*f*(I) = (*y*_N_ − *y*)/(*y*_N_ − *y*_I_),

*f*(II) = (*y*_I_ − *y*)/(*y*_I_ − *y*_U_),

where *y*_N_, *y*_I_, and *y*_U_ are the measured fluorescence intensities of the native, intermediate, and unfolded states, respectively, and *y* is the fluorescence intensity observed at a given *GdmCl* concentration. The unfolded fraction *f*(I or II) data were plotted against *GdmCl* concentrations and theoretical curves, defined by the following equation, were fitted to the experimental data set using a nonlinear curve fitting algorithm (Kaleidagraph 4.5.0, Synergy Software) [60]:

(2) $$f\left(I\;\mathrm{or}\;\mathrm{II}\right)=\frac{\exp\left(-m\frac{\left(C_{m}-\left[GdmCl\right]\right)}{RT}\right)}{1+\exp\left(-m\frac{\left(C_{m}-\left[GdmCl\right]\right)}{RT}\right)},$$

where *T* is the absolute temperature, *R* is the universal gas constant, *C_m_* is the concentration of *GdmCl* at the mid-point of the transition, and *m* the dependence of the Gibbs free energy of the unfolding reaction (Δ*G*) on the denaturation concentration of *GdmCl*. Knowing *C_m_* and *m*, the standard Gibbs free energy of the unfolding reaction in the absence of denaturant, Δ*G^0^_H2O_*, can be calculated according to the relation [58]:

(3) Δ*G^0^_H2O_* = *m C_m_*

### 2.6. 3D-PGM Structure Modelling

PGM 78, 90, and 100 3D protein conformations were modelled using Modeller 9v13 software [61], using the structure of the crystallized rabbit phosphoglucomutase with its substrate, α-D-glucose 1-phosphate, as a template (pdb file 1C47). This protein comprises 561 amino acids with a resolution of 2.70 Å, sharing 65% sequence identity with that of *A. pompejana*. A total of 100 models were generated for each PGM isoform and their quality was assessed using the Modeller Objective Function parameter. Finally, structural optimization was obtained using the repair function of the FoldX software [62].

## 3. Results

### 3.1. Sequencing AP-Pgm-1 cDNA from Homozygous Genotypes

Full-length *Pgm-1* cDNA sequences were obtained from three 100/100 genotypes, three 90/90 genotypes, and only two 78/78 genotypes. This led to a complete cDNA sequence of 562 codons without indels between alleles encoding the three distinct allozymes (Figure S1). The consensus protein sequence fell into the phosphoglucomutase 1 family of proteins, with a blastp e-value of 0.0 (65–72% of identity over 99% of 562 residues with the sequence from the oyster *C. gigas* and a selection of vertebrate species). Of the 16 cloned sequences, only two nonsynonymous mutations on exon 3 allowed us to discriminate the three main allozyme genotypes (i.e., 100/100, 90/90, and 78/78). These polymorphic mutations corresponded to the replacement of a glutamic acid (E) by a glutamine (Q) at positions 155 and 190. Another replacement of a valine (V) by a leucine (L) at position 40 was also found in exon 1 at intermediate frequency, but this amino acid polymorphism was not linked to a given electromorph. The replacement of a phenylalanine (F) by a leucine (L) was also found at position 502 in cDNA encoding allozyme 90.

### 3.2. Assignment of the Two EQ Amino Acid Replacements to Allozymes in Natural Populations

To address the relationship between the two QE substitutions depicted by the cDNA sequences and allozymes, direct sequencing (and/or RFLP) was performed on a portion of exon 3 (94 codons) containing the double diagnostic mutations EQ in 220 individuals from both sides of the EPR, previously genotyped at the PGM-1 enzyme. The linkage disequilibrium between the two EQ mutations (at codon positions 155 and 190) and allozymes was highly significant (Table 1), with correlation coefficients (R_ij_) greater than 70% (*p*-values < 0.0001). This provided very reliable correlations, in which the combinations QE, EQ, and EE corresponded to the isoforms 78, 90, and 100, respectively. The most negatively charged allozyme 112, which is rare and always found at the heterozygous state in the northern populations, was also assigned to genotype EE, suggesting that an additional replacement occurs elsewhere in this protein. From this genotyping, groups of individuals from either the northern or the southern EPR did not depart significantly from the Hardy-Weinberg proportions. However, observed and expected heterozygosities were both greater in the northern population (Ho_-North_: 0.40 vs. Ho_-South_: 0.29). Interestingly, the allele QQ was not found in any of the populations. The frequencies of EE, EQ, and QE alleles at the sampled localities are summarized in Table S2. A more thorough analysis of the north/south genetic differentiation was conducted on the 374 allelic sequences obtained by direct sequencing (see alignment in Supplementary Data). The resulting haplotype network (Figure S2) shows a quasi-complete isolation of the northern and southern populations, with a Fst value of 0.510 (see Table 2). Based on the 282 bp alignment, PGM90 (EQ) found in the southern population derives directly from the northern PGM90 (EQ) by one fixed mutation, while the southern PGM78 (QE) differs by two mutations from the northern PGM100 (EE). The haplotype network also indicated that at least three alleles sampled in the southern populations originated from the northern populations, suggesting that the barrier to gene flow is not complete.

### 3.3. Cryptic Amino Acid Variation along the AP-Pgm-1 Gene

The full sequence of *AP-Pgm-1* with the location of polymorphic codons and primers is shown in Figure S1. The total length of the nucleotidic sequence is 4372 bp. The gene is subdivided into nine exons and eight introns, with lengths ranging from 155 to 848 bp. The coding sequence of 1686 bp (562 codons) has an overall GC content of 43.5% (compared to only 29.3% in the intronic regions). When compared to human and oyster *Pgm-1* genes [63,64], the largest *AP-Pgm-1* exon, comprising 156 codons (other exons vary from 40 to 81 codons), corresponds to the fusion of exons 3, 4, and 5 of the human *Pgm-1*. This fusion is shared with the oyster *C. gigas*, suggesting that annelids and molluscs share the same gene architecture (Figure 2).

**Figure 3:**
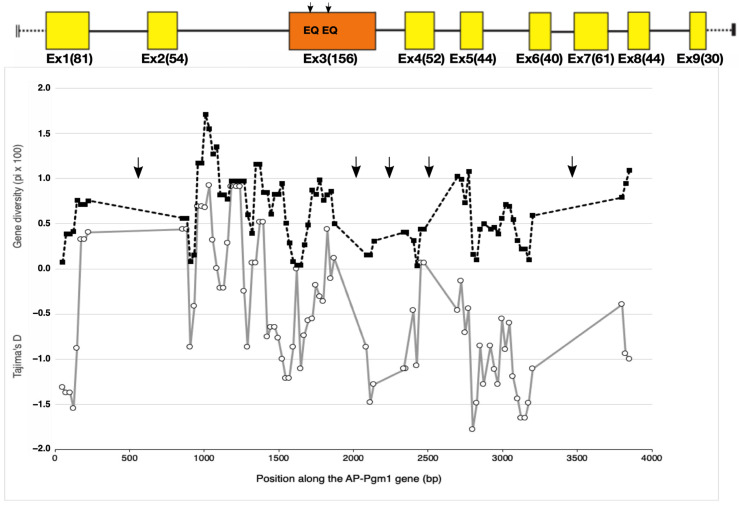
Evolution of gene diversity (π) (black dashed line with black squares) and the Tajima’s D statistic (grey line with white circles) along the *AP-Pgm-1* gene using a sliding window of 100 bp size with a 25 bp step. The analysis includes exonic and intronic fragments for which the sequence polymorphism has been documented. Arrows indicate the portions of the gene for which no genetic data set are available. Black arrows on *AP-Pgm1* exon 3 indicate the positions of the two EQ substitutions.

Besides the two QE changes affecting the net charge of the protein in exon 3, other, less common, cryptic amino acid replacements were found along several regions of the *AP-Pgm-1* CDS. This allowed us to estimate gene diversities and the south/north divergence over an overall portion of about 3 kb (two-thirds of the gene; see Table 2). Gene diversities were almost constant over the *AP-Pgm-1* gene, but allele divergence increased locally in the vicinity of the two segregating EQ sites (Table 2). Looking more closely at the site variation along the gene using a sliding window on our set of sequenced fragments indicated that gene diversity was also slightly higher in exon 3, where the two QE substitutions were found with values almost identical to those depicted in intron 2 (Figure 3). This slight increase corresponded to peaks of positive Tajima’s D values, which increased to +0.5 at the beginning of exon 3, suggesting that the presence of the two linked non-synonymous mutations may be associated with a gene diversity hotspot. However, the observed genetic diversities, as estimated from θ_W_ and π, were not significantly greater than expected from neutral coalescent simulations for both the southern and northern populations over all the investigated *Pgm1* fragments (Figure 3, Table 2). Together with the two QE variant sites, the genotyping of exon 1 also confirmed the occurrence of a trans-equatorial V40L substitution, found at a frequency of 0.15 and restricted to the southern EQ allele (PGM90), and one of the two northern allelic lineages, regardless of the mutations EE (PGM100) and EQ (PGM90). The direct sequencing of the two other genic regions, located either between exons 4 and 5 and between exons 6 and 8, did not show any additional diagnostic amino acid changes between the three allelic lineages EE, EQ, and QE. In contrast, several synonymous changes and indels appeared to segregate between different allelic lineages along the gene (see sequence alignments, provided as supplementary data, for exons 1, 3, 4, 5, and 7 and introns 2, 6, and 7).

### 3.4. Estimating Allele Divergence and Linkage Disequilibria between Segregating Sites

**Figure 4:**
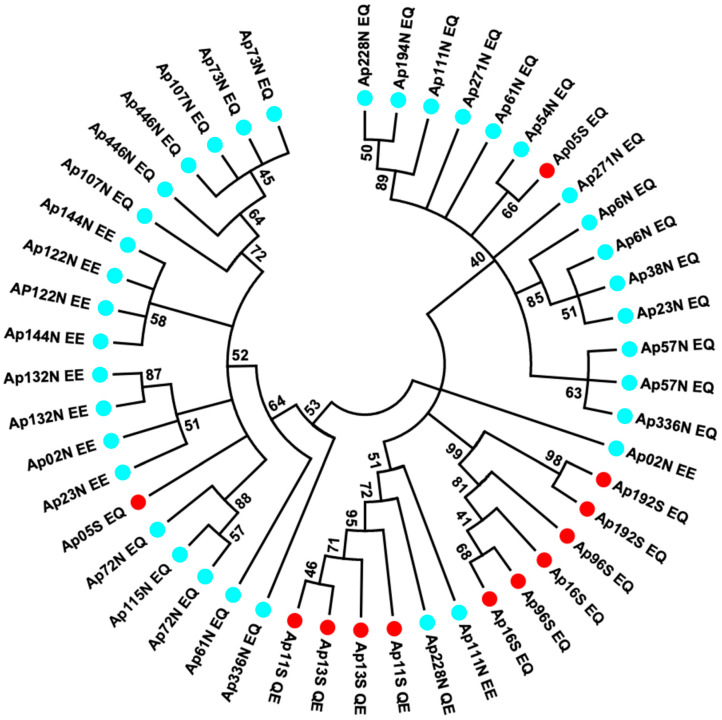
Minimum evolution tree obtained from evolutionary distances computed using the Maximum Composite Likelihood method in MEGA7 on 48 sequences from individuals of the northern and southern EPR locations using the Mark-Cloning-Recapture (MCR) of the *Pgm-1* intron 2 and exon 3 (1110 bp). The sequences corresponding to PGM 78, 90, and 100 are, respectively, identified by the letters QE, EQ, and EE, traducing the polymorphism at positions 155 and 190, with the colours blue and red corresponding to the individuals from the north and south of the EPR, respectively.

To more specifically examine allele divergence and linkage disequilibria between segregating sites within allelic lineages, a sequencing of recaptured alleles was targeted on the longest region of the *AP-Pgm-1* gene (1110 bp). This region, containing intron 2 and the two allozyme-diagnostic sites EQ on exon 3, was genotyped from a subset of individuals. The sequencing of 48 alleles highlighted the presence of a high level of synonymous polymorphism with a strong linkage disequilibrium between these sites (Table 2), as well as two diagnostic indels in intron 2 (insertions referred to as A and B, following their order in the intron). These segregating sites and indels allowed us to determine four divergent allelic lineages with a few recombinants between them. These alleles were split between the northern and southern populations. In the southern population, the two allelic lineages L1 and L2 refer to the allozyme-diagnostic double mutation QE and EQ, whereas allelic lineages L3 and L4 refer to EQ and a mixture of EQ and EE in the northern population (Figure 4). At least nine and eleven synonymous mutations were fixed in intron 2 between allelic lineages L1 and L2, on one hand, and L3 and L4, on the other hand, respectively.

In the southern population, allele L1 was typified by no insertion (QE, no_A, no_B), when compared to allele L2 (EQ and A, B), with a strong linkage disequilibrium between nearly all segregating sites (no recombination, see Table 2). It is worth noting, however, that one individual sampled at 7°25S originated from the northern populations had a L3L4 signature.

In the northern population, the two divergent lineages L3 and L4 also display linked sites, with either the A indel (L3) or the B indel (L4), but these two lineages were not completely associated with the double mutations EE and EQ. Alleles EE were mostly found in one of the two lineages plus one recombinant between L3 and L4, suggesting that these two lineages have recombined once (Figure 4; Table 2). Alternatively, allele EE could derive from one of the two lineages.

To estimate the recombination rate along the gene, we examined the distribution of the *Rho* parameter 4*N.r* with Phase 2.1.1 over a greater proportion of the gene (1–2860 bp), using segregating sites (n = 53) shared between northern and southern individuals that were successfully sequenced for all exon–intron fragments of *AP-Pgm-1* (N = 20). Results from the Phase -recomb and -hotspot outputs clearly indicated that the recombination rate between alleles remained extremely low all along the gene (average local *Rho* = 0.033 and 0.008 for the northern and southern populations, respectively, which increased to nearly 2 in the southern population at the end of the gene, near the position 2140). Our results, therefore, indicated that the four allelic lineages greatly diverged from each other in the vicinity of the double mutation characterizing allozymes, with divergence even greater between allelic sequences in the same population (0.7–1%) than those for the two geographic regions investigated (0.9%).

To test whether the genetic patterns of *AP-Pgm1* may be maintained by selection, population parameters of both the northern and southern populations were simulated using an MSMS structured coalescent with and without selection. Simulations indicated that a low asymmetrical migration across the equatorial barrier with low or no recombination would not explain, by itself, the observed patterns of genetic diversities found for the *AP-Pgm-1* gene (Table S3). Simulated Fst values were around 0.8, and asymmetrical deme diversities were at least two times smaller than the observed ones (π = θ_W_ = 3) with and without recombination. In this context, Tajima’s D was close to zero within each deme, as observed, but highly positive (+2.75) for the overall population when the observed one was also close to zero. Introducing selection led to a better fit of the simulated parameters to the observed ones. Simulations with over-dominance and low recombination led to a slight decrease in Fst values (0.7) between demes, as well as an increase in the within-deme genetic diversities close to the observed ones, but also produced greater positive Tajima’s D (+1.3 for each deme). Our best fit to values observed in the worm populations was obtained with the two-niche model simulations (Fst = 0.45; nucleotidic diversities θ_W_ and Tajima’s D estimates converging respectively, from 13 and +0.4 to 17 and +0.8 within and between demes). These simulated values were even closer to those estimated in the vicinity of the two EQ sites (intron 2; see Table 2 and Table S3).

### 3.5. Conformational Stability, Thermal Inactivation, and Kinetics of the Mutated Isoforms

**Figure 5:**
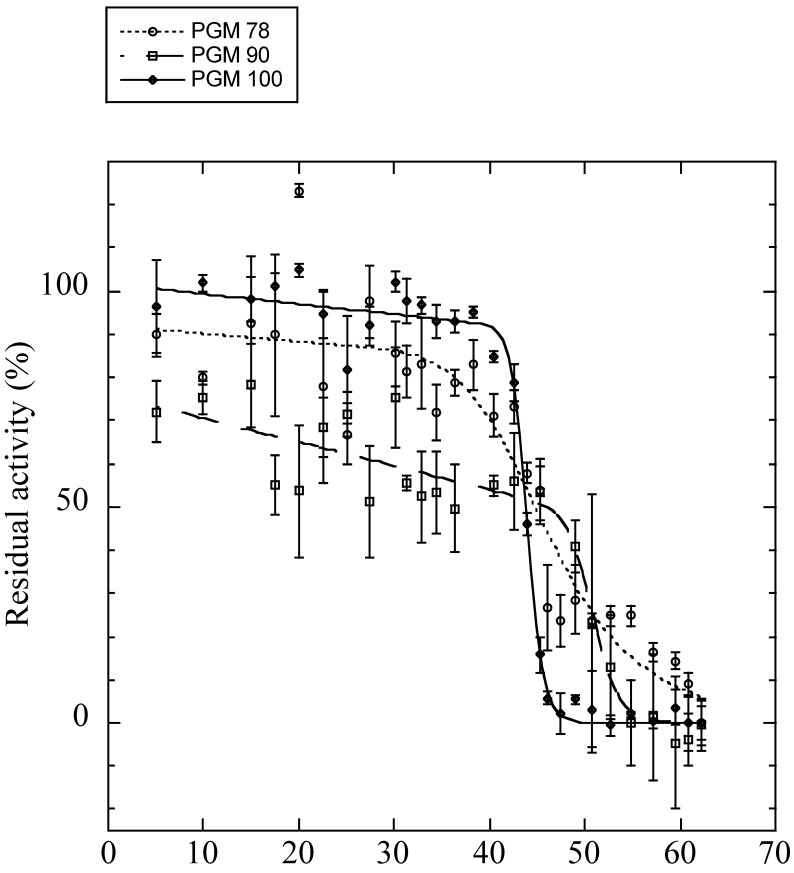
Residual enzyme activities after 30 min of incubation at different temperatures (on x-axis) for the expressed isoforms of PGM 78 (QE: dotted line with white circles), 90 (EQ: dashed line with white squares), and 100 (EE: plain line with black diamonds). *T_m_* values are provided in Table 3.

Obtaining full-length *AP-Pgm-1* cDNAs allowed us to examine the direct effect of the two QE substitutions on the thermal stability and efficiency of the PGM-1 enzyme using in vitro directed mutagenesis. To determine the conformational stability of the three recombinant isoforms of PGM-1, their guanidinium chloride (*GdmCl*)-induced unfolding was studied. Variations of fluorescence with increasing concentrations of *GdmCl* were biphasic (Figure S3), suggesting that the protein follows a three-state model of denaturation. For each transition, the unfolded fraction of protein (*f_u_*) was determined (Figure S4) and the Gibbs free energy change associated with each transition was calculated (Table 3). For the two transitions, PGM90 (EQ) appeared more stable than the two other isoforms. PGM78 (QE) appeared more stable than PGM100 for the first transition (Δ*G^0^_H2O_* = 8.0 ± 0.46 kJ.mol^−1^ vs. Δ*G^0^_H2O_* = 6.06 ± 0.81 kJ.mol^−1^), but not for the second transition (Δ*G^0^_H2O_* = 15.43 ± 0.93 kJ.mol^−1^ vs. Δ*G^0^_H2O_* = 15.13 ± 0.98 kJ.mol^−1^). The *T*_m_ values obtained from the theoretical curve fitted to the thermal inactivation experimental data (Figure 5, Table 3) were very similar for PGM78 (46.5 ± 1.7 °C) and PGM100 (44.0 ± 0.1 °C), but markedly higher for PGM90 (50.9 ± 0.7 °C). 

Enzyme kinetic analyses of the three PGM isoforms are also presented in Table 3. The catalytic efficiency of PGM78, evaluated by the ratio *k*_cat_/*K^app^_m_*, appeared to be 125 and 70-fold higher than that of PGM90 and PGM100, respectively. Changes in both *K_m_^app^* (for the substrate Glucose 1 phosphate, G1P) and *k*_cat_ explained most of the difference in the catalytic efficiency of the three isoforms. *K_m_^app^* (G1P) and *k*_cat_ of PGM78 were, indeed, tenfold lower and tenfold higher than that of the two other isoforms, respectively (see Table 3).

### 3.6. Fitness Cost of Individuals Carrying the Thermostable Allele in Terms of Female Fecundity

The Pompeii worm females exhibited an average coelomic fecundity of 200,000 oocytes, with great variability among them (values ranged from 1200 to 450,000 oocytes, depending on size, age, and reproductive status [42]). As opposed to our expectations, females carrying the allele 90 were, on average, more fecund than homozygous females carrying alleles 78 and 100; however, distributions of fecundity corrected by the size of the female did not significantly differ between *Pgm-1* genotypes (One-way ANOVA: F = 1.08, *p* = 0.37; see Figure S5). This finding clearly indicates that the ability to live under hotter conditions is not counter balanced by lower reproductive success; at least, for the females.

## 4. Discussion

Based on allozyme data, Piccino et al. [12] previously proposed that the enzyme polymorphism of the Pompeii worm *A. pompejana* may be balanced at the locus *Pgm-1*, at least in populations of the northern EPR. They showed that allozymes 90 and 100, indeed, display distinct thermal stabilities and kinetic optima, with the frequency of the most thermostable isoform (allozyme 90) being positively correlated with temperature in newly formed edifices [12,36]. As the Pompeii worm is the only vent species capable of colonizing newly formed, still-hot hydrothermal chimneys, bearing thermostable alleles is likely to represent an adaptive advantage. The maintenance of polymorphism by selection on thermostable alleles, however, remains unresolved. If advantageous in the hottest part of the vent environment, thermostable alleles can be expected to spread rapidly in the population through recurrent selective sweeps. This is obviously not the case for the *Pgm-1* locus, which exhibited three major isoforms of different thermal stabilities (allozymes 78, 90, and 100), showing sharp frequency differences across the equatorial barrier to gene flow, as depicted by Plouviez et al. [40]. Several hypotheses have thus been proposed by Piccino et al. [12], regarding the maintenance of alleles at the PGM-1 enzyme. These includes: (i) allele over-dominance due to the rapid alternation of aerobic/anaerobic vent conditions; (ii) a fitness cost for individuals carrying the most thermostable allele at this locus; and (iii) a two-niche model effect, due to fluctuating proportions of ‘hot’ and ‘cold’ habitats along the EPR.

In the present study, we sequenced the three major *Pgm-1* alleles of the worm to investigate the distribution of non-synonymous polymorphisms along the gene, examine their relationship with allozymes, and assess their evolutionary fate. We demonstrated that only two linked mutations (E_155_Q and E_190_Q) are associated with the net charge of allozymes and are also responsible for the thermal performance of the three allozymes (78, 90, and 100). Examining the evolutionary history of these alleles indicated their rather old origin, which predates the vicariant event that separated the EPR vent fauna across the Equator about 1.2 million years ago [40,65,66]. In the following discussion, we therefore examine arguments towards the adaptive maintenance of PGM-1 isoforms. We propose that thermal compensation represents a powerful mechanism by which different enzymatic properties might be maintained under balancing selection—at least, during the exploration of the mutational landscape of the protein that will lead to the emergence of the ‘optimal’ isoform—in order to optimize metabolic fluxes, as previously stated by Eanes [67].

### 4.1. Two-Allele Polymorphism at the Pgm Locus: A Long Story of Balancing Selection?

The non-synonymous polymorphisms associated with the four allelic lineages of *AP-Pgm-1* appeared to be quite low (π_N_ = 0.0025, on average). Such a result sharply contrasts with earlier studies on branch-point glycolytic enzymes (that control the metabolic flux for transport, storage, and breakdown of carbohydrates), for which numerous cryptic non-synonymous changes were described [67]. High levels of gene diversity have indeed been observed between the slow, medium, and fast electrophoretic *Pgm-1* alleles of *Drosophila melanogaster* [14,68], and between phosphoglucose isomerase (PGI) alleles of *Colias* butterflies [69], suspected to evolve under balancing selection. By contrast, only eight nonsynonymous mutations (E37Q, V40L, E155Q, E190Q, R343I, G358S, T366M, and F502L), some of which were at low frequency, were detected by the direct comparison of allelic sequences of *A. pompejana*. Moreover, even if gene diversity was slightly higher in the intronic region preceding exon 3 and the exon 3 itself where the two EQ sites are found, its variation along the gene does not fit perfectly with the expectations of long-term balancing selection. A weak hotspot signal of silent site variation, supported by slightly positive Tajima’s D and Fu & Li’s F statistics was, however, observed near the doubly selected sites E^155^Q/E^190^Q, but not as strong as signals depicted for the *Adh* locus in *Drosophila* [70,71]. Theoretical effects of balancing selection on nearby genome regions promote increase in genetic diversity near the selected site due to lack of recombination and the long-term accumulation of mutations [38]. The very low level of nucleotidic polymorphism found at *AP-Pgm-1* can, however, be partially explained by recurrent population bottlenecks, due to the challenging environmental conditions that affect the whole vent fauna [15]. The joint action of abrupt demographic changes and habitat specialization should, indeed, promote enzyme monomorphism. Under such conditions, the level of polymorphism observed at *Pgm-1*, although low, appears to be quite unusual when compared to most of the genes examined in *A. pompejana*. In alvinellid worms—and, especially, thermophilic species—proteins are under strong purifying selection, with transcriptome-wide d_N_/d_S_ averages very close to zero, and individual gene values ranging between 0.02 and 0.05 [72]. To this extent, it is worth noting that gene diversity at the *Pgm-1* locus appears to be locally two to four-fold higher than the genome-wide average value (ddRAD overall π = 0.0025 [73]), and from other reported genes [35].

Looking more specifically at the four allelic lineages of *AP-Pgm1* near the EQ sites (intron 2) clearly indicates that they have accumulated a great number of synonymous substitutions due to their separation with almost no recombination events (low values of *R_m_* and *Rho*, see the Phase 2.1.1 analysis). The two allelic lineages, L1 and L2, present in the southern population exhibited 1% divergence between them, with a strong linkage disequilibrium between the two variant sites E^155^Q^190^ (PGM90) and Q^155^E^190^ (PGM78), and the silent substitutions found in intron 2. This suggests that the two allelic lineages evolved separately, without recombination in the vicinity of the two nonsynonymous sites, for a long period of time. The two northern allelic lineages (L3 and L4) also diverged by 0.7% and display two diagnostic indels in intron 2. These silent mutations and indels are also linked, suggesting once more that these two lineages evolved separately, but for a shorter period of time. However, diagnostic mutations were not completely linked to the variant sites E^155^E^190^ (PGM100) and E^155^Q^190^ (PGM90). Although L3 forms a single clade associated with E^155^Q^190^, L4 is a mixture of E^155^E^190^ and E^155^Q^190^, suggesting either that at least one recombination event occurred between the two northern alleles, or that E^155^E^190^ (PGM100) is a recently derived variant in the northern population. Finally, both divergences observed between alleles within each population were of the same amplitude as the divergence estimated between the southern and northern alleles (0.9%), which coincided with an overall significant F_st_ value of 0.588 between them. If we accept that the southern (L1 and L2) and northern (L3 and L4) allelic lineages became isolated after the appearance of the physical barrier to dispersal—about 1.2 million years ago [35,40]—this clearly indicates that the co-occurrence of the four highly divergent *Pgm-1* alleles derives from an older polymorphism, predating the vicariant event that separated the northern and southern vent fauna of the East Pacific Rise, with the possible emergence of the genotype E^155^E^190^ in the north. Such a scenario is likely confirmed by the distribution of alleles in the exon 3 haplotype network and the signature of the linked silent polymorphic sites in introns 1 and 2, where northern alleles seem to derive from a southern allele bearing the Q^155^E^190^ mutation.

### 4.2. Selective Modalities for the Maintenance of a Balanced Polymorphism

The long-term evolution of *Pgm-1* alleles without recombination—at least, in the first part of the gene—and their frequency changes according to environmental conditions, raises questions regarding the selective modalities acting on the co-occurrence of alleles when one of the two alleles is better adapted to high temperatures [12]. One of the first explanations for the adaptive maintenance of AP-PGM1 isoforms was to consider that the rapid alternation of oxic and anoxic conditions during venting should favour heterozygote excesses if the heterozygote’s fitness is close to that of the favoured homozygote in one of the two habitats [74]. Pogson [13] previously proposed that over-dominance represents the most likely evolutionary mechanism at the origin of the maintenance of a balanced polymorphism at the *Pgm-2* locus for the oyster *C. gigas* (see also [75], for its link with the individual’s growth rate). Here, we were not able to detect any overdominance at the *Pgm-1* locus in terms of heterozygote excesses in natural populations. Simulations of structured coalescent with asymmetrical migration and over-dominance always led to very high within-deme positive Tajima’s D and unequal diversities between overall and within demes (not found in the observed dataset). This finding confirms a previous study [12] considering allozymes. However, it is worth noting that such an advantage can be easily masked by the temporal dynamics of the thermal habitat (i.e., chimneys refreshing with time) and the juxtaposition of chimneys of different ages. The worm is, indeed, exposed to a mosaic of fluctuating thermal habitats where temperature could vary spatially, according to the age of the chimneys [12].

Maintenance of allozymes with different thermal stabilities can be also explained by a two-niche model of local differentiation between habitats and drift [76,77,78,79]. Simulating coalescences with a two-niche model and a theta value equal to the observed value provided parameter estimates (Fst, as well as diversities and Tajima’s D) much closer to values observed in the vicinity of the two EQ sites than the two other models of asymmetric gene flow with and without over-dominance. Given the spatial and temporal dynamics of the hydrothermal discharge, changes in the frequency of *Pgm-1* alleles could be either due to local selection or exacerbated genetic drift associated with the dynamics of colonization of the newly opened sites. Indeed, the dynamic nature of hydrothermal vents over longer time scales (i.e., years) led to a very patchy and transient habitat scattered along the EPR, with a complex heterogeneity of age-driven vent conditions. This can be seen as a multitude of distinct ecological niches for the same species. In this context, the proportion of newly formed ‘still hot’ chimneys and older ‘colder’ ones greatly vary over time. These dynamics depends on the spreading rate of the rift and, thus, the frequency of tectonic and volcanic events along the East Pacific Rise.

To explain the maintenance of a bi-allelic polymorphism at the *Pgm-1* locus, Piccino et al. [12] proposed a fitness cost of worm colonization during the early stages of a chimney. The first settlers on ‘hot’ (>100 °C) anhydrite chimneys benefit from a lack of predators and competitors. Colonists may, therefore, display more thermoresistant alleles but lower reproductive investment and/or survival. Watt et al. [80,81,82] and Wheat et al. [69] previously showed that both PGI and PGM have a great contribution to the mating fitness of male *Colias* butterflies, likely as the result of longer and more vigorous flight within the day. However, based on female fecundity, we were not able to observe fitness differences between the *Pgm-1* genotypes. This suggests that a better predisposition to colonize still ‘hot’ chimneys is probably not compensated by reduced reproductive success to prevent fixation of the advantageous allele. In fruit flies, the *Pgm* locus represents a quantitative trait for glycogen storage and, hence, the ability to survive better under starvation [34]. In the case of *A. pompejana*, differences in the thermal regime could be great between colonists and reproducers. As a consequence, colonists subjected to longer periods of high temperature (and associated hypoxia) may be maladapted to produce and use their glycogen reserves. This should have consequences in their investment into reproduction. To the contrary, secondary settlers arriving in much cooler conditions are more likely to use their glycogen reserves to massively invest in the production of gametes, as was previously shown by Faure et al. [42]. Colder conditions seem to be a prerequisite for releasing fertilized eggs after pairing, as embryos are not able to develop at temperatures greater than 15 °C [41].

### 4.3. Adaptive Polymorphism: A Trade-Off between Enzyme Thermostability and Catalysis

In *Drosophila*, *Pgm-1* variants play a non-negligible role in regulating the metabolic energy pool along latitudinal clines, where a decrease in temperature is compensated for by an increase in enzyme activity. Populations of *D. melanogaster* living at the highest (and, thus, coldest) latitudes possess PGM allozymes with a higher catalytic efficiency and greater glycogen contents. According to the theory of metabolic flux, this can be an adaptive means of temperature compensation to maintain the same glycogen contents over the latitudinal gradient [67]. Differences in both protein thermostability and catalytic efficiency between *Pgm* alleles have been previously reported to explain both local differentiation and latitudinal clines in the oyster *C. gigas* [6,13] and *D. melanogaster* [34]. By comparison, thermal compensation may be directly linked to different biochemical phenotypes that interact with the growth rate and reproductive effort of the worms. The theory predicts that differences in activity at only one enzyme must be substantial to affect metabolic fluxes between genotypes [83]. To test this hypothesis, one could measure the effect of non-synonymous mutations on the functional properties of the enzyme, its conformational stability, and their effects on population fitness. In this study, three recombinant isoforms of PGM-1—E^155^E^190^ (PGM100), E^155^Q^190^ (PGM90), and Q^155^E^190^ (PGM78)—were obtained by directed mutagenesis. The replacement of the glutamate by a glutamine at position 190 increased the conformational stability and thermostability of the protein, confirming that PGM90 is the most thermostable isoform. As discussed by Piccino et al. [12], carrying this allele may be advantageous during the colonization of newly formed chimneys, whose surface temperature usually exceeds 50 °C. Unexpectedly, PGM90 exhibited a decrease in the catalytic efficiency of the enzyme when compared to the two other recombined variants (*kcat/Km* was a hundred times for PGM78 and nearly two-fold greater for PGM100, when compared to PGM90). The recombinant PGM90 also exhibited the lowest affinity for its substrate, glucose-1-phosphate, at 17 °C. This finding is of importance, as such a genetically determined trade-off between protein stability and enzyme activity has not, so far, been reported in other invertebrate species subjected to balancing selection [6,13,34,82]. Increased thermostability of a protein is often associated with a decrease in the flexibility of the molecule and, thus, the dynamics of the enzyme reaction [84,85]. Our results are in perfect agreement with these theoretical expectations. They support the positive role of a thermodynamic trade-off between thermostability and catalysis, as previously proposed by Eanes [11], to explain the co-occurrence of alleles. PGM90 can remain stable for a longer period of time but is less efficient in either producing or consuming the glycogen reserves of the worm than the two other isoforms. To this extent, the fact that the *k_cat_/K_m_* ratio of isoform PGM78 is much higher than that of the isoform PGM100 can explain why isoform 78 is more frequent in the southern populations (about 80%), when compared with isoform PGM100 in the northern populations (around 70%). The balance between allele frequencies from both sides of the Equator may be dictated by the selective coefficient attributed to each genotype as a direct reflection of the catalytic efficiency difference between PGM90 and its alternative isoforms.

### 4.4. Structural Effect of Mutations E155Q and E190Q

**Figure 6:**
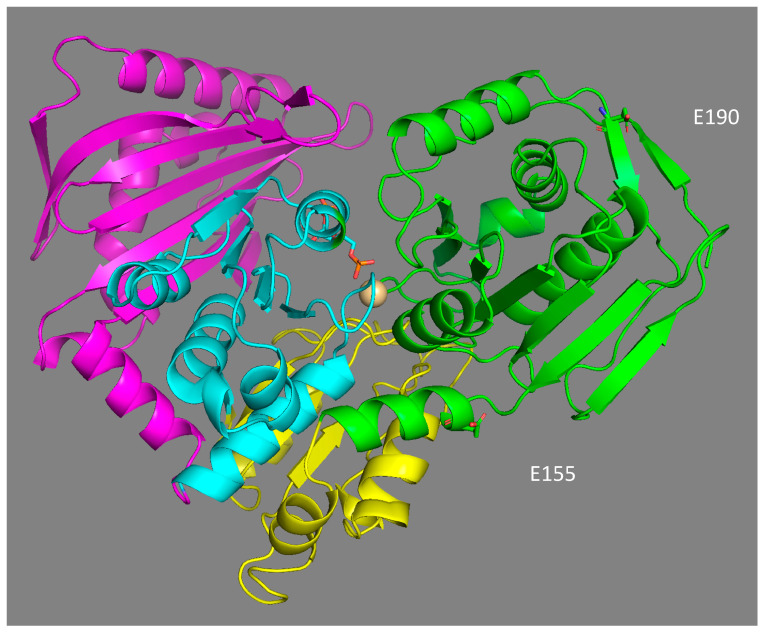
3-D structural model of *A. pompejana* PGM 78 fitted to the PGM-1 rabbit template (1C47, 2.70 Å) using Modeller 9v13 software. The protein is structured in four domains, labelled from I to IV (I green, II yellow, III blue, IV violet). Positions 155 and 190 of EQ replacements belong to domain I near the catalytic site of the enzyme, which binds the reaction catalyser, α-D-glucose-1,6-diphosphate, and the Mg^2+^ ion.

The location of the two main polymorphic sites (E155Q and E190Q) in the 3D model structure of phosphoglucomutase 1 expose them to solvents, and are not located in the binding domains of the enzyme (Figure 6). Their potential effect on the catalytic properties of the enzyme is, therefore, not the result of a direct interaction with the substrate and/or the residues involved in catalysis. This is not surprising, as most of the mutations affecting the binding of the substrate glucose-1-phosphate, the Mg^2+^ ion, and phosphate should be deleterious. Similarly, in a study of the polymorphism of the enzyme PGM in *D. melanogaster*, none of the 21 polymorphic amino acid replacements were located in the catalytic site of the enzyme [34]. Based on their location, both substitutions should affect the net charge of the protein in the same way. However, the isoforms 78 and 90 did not have the same electrophoretic mobility, suggesting that some post-translational modification may be involved in the electrophoretic separation of the three isoforms. The gain of a glutamate at position 155 may be associated with a potential ionic bond with histidine 157, with a distance of 6.5 Å between them. Ionic and hydrogen bonds have been shown to increase the stability of enzymes [86] and partially explain the thermostable 3D structure of the Cu–Zn and Mn superoxide dismutase enzymes in *A. pompejana* [87,88]. This may account for the increased thermostability of isoform 90 but should also have the same effect for isoform 100, which was obviously not the case. This suggests a negative effect of glutamate (E) at position 190, which negates the positive effect of glutamate at position 155. Alternatively, the 3D model comparison of the three isoforms shows that the replacement of one glutamine (Q) by a glutamate (E) at position 190 (as in allozymes 78 and 100) introduces a negative charge in a region already enriched in acidic residues. This high density of negative charges could have a destabilizing effect on the protein structure through a Coulomb repulsion effect, thus potentially leading to greater sensitivity to temperature. Finally, the glutamine replacement at position 155 is also likely to play a key role in the molecular dynamics of the protein, especially during the 180° rotation of the reaction intermediate (glucose-1,6-diphosphate) inside the active site. This potentially explains the higher enzymatic efficiency of the isoform 78.

## 5. Conclusions

Exploration of the mutational landscape of phosphoglucomutase 1 revealed the maintenance of four divergent allelic lineages over the geographic range of *A. pompejana*. The enzyme polymorphism, only governed by two linked amino acid replacements (E155Q and E190Q), is likely to be maintained by balancing selection, and better fits with a two-niche model of selection, in which ‘cold’ and ‘hot’ conditions alternate. These isoforms do not seem to be maintained by higher reproductive fitness of females that do not carry the thermostable allele needed during the colonisation of new ‘hot’ chimneys. This persistence over a long period of time may rather be explained by a thermodynamic trade-off between protein thermostability and catalysis of these functional phenotypes.

## Figures and Tables

**Table 1:** Linkage disequilibrium between the combination of the two diagnostic mutations EQ and PGM-1 allozymes.

Mutation	D_ij_	R_ij_	Chi^2^	*p*-Value
EE-100	0.274	0.908	87.2	0.0001 ***
EQ-90	0.115	0.725	55.7	0.0001 ***
QE-78	0.357	0.907	87.2	0.0001 ***
EE-112	0.013	0.230	5.6	0.0178 *

*: *p* = 0.05; ***: *p* = 0.001.

**Table 2:** Gene diversities, population parameters, and neutrality tests along the *Pgm-1* gene for *A. pompejana* populations from the southern and northern EPR.

Statistics	E1 North	E1 South	E2-I2 North	E2-I2 South	E3 North	E3 South	E4-E5 North	E4-E5 South	E7-E9 North	E7-E9 South
Fragment length (bp)	273	273	1110	1110	278	278	803	803	576	576
N	38	40	36	12	156	218	20	45	62	12
H_d_	0.77 ± 0.06	0.39 ± 0.01	0.95 ± 0.03	0.85 ± 0.02	0.72 ± 0.03	0.76 ± 0.04	0.68 ± 0.10	0.88 ± 0.04	0.91 ± 0.03	0.98 ± 0.04
_Overall_ π	0.0059 ± 0.0006	0.0017 ± 0.0005	0.0056 ± 0.0003	0.0087 ± 0.0005	0.0045 ± 0.0003	0.0070 ± 0.0006	0.0023 ± 0.0005	0.0045 ± 0.0005	0.0044 ± 0.0005	0.0068 ± 0.0010
π _s_	0.0016 (62.6)	0.0008 (62.6)	0.0126 (58.7)	0.0404 (58.7)	0.0034 (48.7)	0.0167 (48.7)	0.0094 (88.1)	0.0164 (88.1)	0.0000 (27.8)	0.0000 (27.8)
π _n_	0.0029 (210.4)	0.0020 (210.4)	0.0027 (205.3)	0.0056 (205.3)	0.0030 (170.3)	0.0028 (170.3)	0.0003 (307.9)	0.0012 (307.9)	0.0018 (110.2)	0.0028 (110.2)
S	8	5	26	26	16	18	5	10	24	13
θ_W_ (S)	0.0070 ± 0.0031	0.0043 ± 0.0022	0.0057 ± 0.0033	0.0078 ± 0.0042	0.0102 ± 0.0026	0.0102 ± 0.0026	0.0036 ± 0.0022	0.0058 ± 0.0024	0.0094 ± 0.0030	0.0076 ± 0.0035
Z_n_S	0.054 (1/15/1 ^B^)	0.008 (0/1/0 ^B^)	0.110 (43/325/19 ^B^)	0.466 (46/153/0 ^B^)	0.0093 (2/36/1 ^B^)	0.0540 (11/66/7 ^B^)	0.0226 (0/3/0 ^B^)	0.0324 (3/21/0 ^B^)	0.0261 (4/120/1 ^B^)	0.1641 (5/36/0 ^B^)
R_m_	1 (RDP n.d.)	0 (RDP n.d.)	4 (RDP = 1)	0 (RDP n.d.)	3 (RDP n.d.)	6 (RDP n.d.)	0 (RDP n.d.)	3 (RDP n.d.)	5 (RDP n.d.)	2 (RDP n.d.)
F_st_	0.256 ***		0.262 ***		0.510 ***		0.291 **		0.015 *	
D_xy_	0.0051		0.0098		0.0117		0.0059		0.0059	
Tajima’s D	−0.46 ^NS^	−1.52 ^NS^	−0.07 ^NS^	+0.46 ^NS^	−1.57 ^NS^	−1.12 ^NS^	−1.07 ^NS^	−0.66 ^NS^	−1.73 ^NS^	−0.41 ^NS^
Fu & Li’s F	−0.19 ^NS^	−1.89 ^NS^	+0.07 ^NS^	+0.34 ^NS^	−2.31 *	−1.15 ^NS^	−0.69 ^NS^	−0.57 ^NS^	−1.40 ^NS^	+0.07 ^NS^

N and S represent the number of sequences and the number of segregating sites used, respectively. Linkage disequilibria between sites were estimated between informative sites only: numbers in brackets correspond to the number of significant exact Fisher tests, total number of comparisons, and number of tests still significant after the Bonferroni correction, respectively. (RDP n.d.): Recombinant not detected using automated RDP and bootscan packages of RDP v.3.44. Values in brackets below π _s_ and π _n_ (Jukes & Cantor estimates) are the numbers of synonymous and nonsynonymous sites in coding regions, respectively. All genetic data sets obtained using the MCR method were corrected for artefactual/somatic singletons. *: *p* = 0.05; **: *p* = 0.01; ***: *p* = 0.001. ^NS^: Non Significant. ^B^: Bonferroni test.

**Table 3:** Conformational and temperature stability of the three expressed variants PGM78, PGM90, and PGM100.

	PGM 78		PGM 90		PGM 100	
	1st Transition	2nd Transition	1st Transition	2nd Transition	1st Transition	2nd Transition
*C_m_* (M)	0.50 ± 0.01	2.32 ± 0.02	0.53 ± 0.02	2.42 ± 0.05	0.41 ± 0.02	2.31 ± 0.03
*m* (kJ.mol^−1^.M^−1^)	16.00 ± 0.88	6.65 ± 0.39	21.62 ± 3.73	7.48 ± 1.13	10.45 ± 1.28	6.55 ± 0.42
Δ*G^0^_H2O_* (kJ.mol^−1^)	8.00 ± 0.46	15.43 ± 0.93	11.46 ± 2.03	18.10 ± 2.76	6.06 ± 0.27	15.13 ± 0.98
*Tm* (°C)	46.5 ± 1.7		50.9 ± 0.7		44 ± 0.1	
*K^app^_m_* (mM)	0.76 ± 0.07		6.25 ± 0.35		5.22 ± 0.74	
*K_cat_* (sec^−1^)	192 ± 3.3		12.7 ± 0.5		18.3 ± 0.2	
*K_cat_/K^app^_m_* (sec^−1^.M^−1^)	252.63 ± 17.06		2.0 ± 0.1		3.51 ± 0.49	

*Cm* and *m* values estimated from the variation of protein fluorescence in the presence of an increasing concentration of GdmHCl (values for each of the two transitions). Estimation of the free enthalpy of the unfolding reaction in the absence of the chaotropic agent for each of the two transition states. *Tm*: values of the temperature at which we reach 50% of non-reversible inactivation after a 30-min exposure. *K^app^_m_* and *K_cat_* are kinetic parameters corresponding to the apparent Michaelis-Menten constant for glucose-1-phosphate and the catalytic constant, respectively. The ratio of these two values corresponds to the specific activity.

## Data Availability

Not applicable.

## Supplementary Materials

The following are available online at https://www.mdpi.com/article/10.3390/genes13020206/s1. Figure S1: Entire sequence of the *Pgm-1* gene of *A. pompejana* and its translated exons. Figure S2: Haplotype network obtained by genotyping of the *Pgm-1* exon 3 on 187 individuals coming from the northern and the southern EPR. Figure S3: Guanidium chloride (*GdmCl*) denaturation curves for the three PGM1 expressed isoforms. Figure S4: Modelled regression curves of the folded/unfolded protein states *f_u_*(I) and *f_u_* (II) of three PGM1 expressed isoforms for each transition according to the denaturation equilibrium N↔I↔U (Native↔Intermediate↔Unfolded). Figure S5: Scatterplots of female *A. pompejana* fecundities according to their PGM-1 allozyme genotypes. Table S1: Sequence of the primer pairs, and their use in the study. Table S2: Frequencies of EE, EQ and QE *Pgm-1* alleles, heterozygosities and Fis (*: Significant with 1000 permutations) in northern and southern populations of *A. pompejana*. Table S3: Summary statistics of structured coalescent simulations (*n* = 1000) obtained with MSMS software and the pylibseq libraries in order to test a model of asymmetric migration across a barrier with and without selection and two levels of recombination in order to examine the genetic expectations of overdominance and the two-niches (two demes/two habitats) models.
